# A cross-sectional study of health and well-being among newly settled refugee migrants in Sweden–The role of health literacy, social support and self-efficacy

**DOI:** 10.1371/journal.pone.0279397

**Published:** 2022-12-19

**Authors:** Maissa Al-Adhami, Erik Berglund, Josefin Wångdahl, Raziye Salari

**Affiliations:** 1 Research and Learning for Sustainable Development and Global Health (SWEDESD) Department of Women’s and Children’s Health, Uppsala University, Uppsala, Sweden; 2 Child Health and Parenting (CHAP), Department of Public Health and Caring Sciences, Uppsala University, Uppsala, Sweden; 3 Department of Public Health and Caring Sciences, Uppsala University, Uppsala, Sweden; 4 Department of Clinical Neuroscience, Karolinska Institutet, Stockholm, Sweden; 5 Aging Research Center, Karolinska Institutet & Stockholm University, Stockholm, Sweden; Universität Wien: Universitat Wien, AUSTRIA

## Abstract

Structural barriers such as inadequate housing, lack of employment opportunities, and discrimination are known to adversely affect the health of newly settled refugee migrants. However, these barriers remain largely unresolved and unaddressed. Thus, there is a need to better understand how other factors, such as individual-level health resources, may influence health and mitigate ill health in the early post-migration phase. In this study, we aimed to explore the relationship between health outcomes and individual health resources including health literacy, social support, and self-efficacy in newly settled refugee migrants. Survey data was collected from 787 refugee migrants in Sweden. Logistical regression analysis showed that limited health literacy, lack of emotional support, and low self-efficacy were consistently associated with poor health outcomes. Demographic variables such as gender, education, and type of residence permit were not as imperative. Individual-level health resources may play an important role in the general and psychological well-being of newly settled migrants. Promoting health literacy and facilitating the attainment of social support may buffer for structural challenges in the establishment phase and enhance the prospects of later health and social integration.

## Introduction

Sweden is one of the largest recipient countries of refugee migrants in the European Union (EU). At the end of 2018, Sweden had 25 refugees per 1,000 inhabitants compared to the average of 2.7 per 1,000 in receiving high-income countries [1]. In recent years, both number of granted asylum applications and new arrivals have dropped markedly due to new immigration policies and the effect of the Covid-19 pandemic. Nevertheless, even with the implementation of restrictive immigration policies in Sweden and other EU countries, the WHO estimates that one in 10 persons in the European Region is a migrant [2], making the issue of migrants and refugees health a major public health concern.

The health of refugee migrant populations is often poorer than among native-born populations in the receiving high-income countries. This has been shown for both general self-rated health (SRH) [3] and mental health [4–6], in the early stages of the resettlement process as well as beyond [7, 8]. Health is affected by factors that occur throughout the migration process, i.e., pre-, peri- and post-migration [9]. Differences in pre-migration health and experiences in the migratory process are important to consider in the post-migration phase as they can explain differences in health outcomes within the migrant group, e.g., mental health outcomes [6, 10]. However, factors in the post-migration resettlement process are increasingly being recognized as important for migrants’ health [11–13] as they are, on one hand, often modifiable, and on the other hand, affect migrants as a group, irrespective of prior health.

With regard to post-migration factors that negatively impact migrants’ health, numerous studies have reported the importance of socio-political factors such as uncertainty about asylum processes [14, 15], socio-economic factors such as housing and unemployment [4, 16], and contextual factors such as isolation and discrimination [11, 17]. Although these structural factors have a large impact on health [18] (arguably the largest) and as imperative as it is to address them, they remain largely unaddressed and unresolved in many receiving countries. This gap justifies investigations of factors that may increase resilience at the individual level, e.g., how different health resources may be related to the newly settled migrants’ health and well-being, as well as their potential to buffer against post-migration barriers. We define individual health resources as different forms of downstream and midstream social determinants of health, e.g., health-related knowledge, abilities, and social support [19, 20]. The concept is inspired by the Ottawa Charter for Health Promotion where health is defined as social and personal resources, as well as physical capacities [21]. Specifically, we examine (1) health literacy, i.e., people’s knowledge, motivation, and competencies to access, understand, appraise, and apply health information [22], (2) social support, i.e., sharing, trusting and aiding relationships and networks between individuals that share a common social identity [23, 24], and (3) self-efficacy, defined as an individual’s belief in their ability to cope with and act on challenging demands and situations [25].

Migration is likely to affect these resources adversely. Newly settled migrants are particularly vulnerable to loss of health literacy as they are faced with a new context and system. The same loss applies to social support as they have left social networks in their countries of origin and are yet to establish new ones [26]. Further, in the early resettlement phase, the vulnerability of the newly settled is amplified by the need and demands to adapt to, navigate and interact with new societal institutions in a new context [27] while learning the language and finding employment. The efficacious outlook that aided migrants to reach their destinations could decrease in face of post-migration challenges. It has been theorized that limited health literacy, social support, and self-efficacy may adversely impact the resettlement process as they are determinants of health [19] linked to empowerment in general [22, 24, 25]. Reversely, as these factors are modifiable, they may buffer for experienced difficulties and mitigate ill health post-migration [28]. Thus, a better understanding of individual factors that influences health in the early post-migration phase could improve the life situation of refugee migrants as well as future health and integration.

Previous studies have shown that health literacy and social support and capital are low in migrant populations and associated with poor self-rated and mental health [6, 7, 29–31]. As for self-efficacy, previous studies have found low self-efficacy to be associated with poor health outcomes among refugees and migrant populations [26, 32]. However, comparatively fewer empirical studies have focused on the effect of health literacy, social support, psychological well-being, and self-rated health of migrants in the early post-migration phase [7, 31, 33], defined here as having spent less than five years in the country of settlement. Using ‘individual health resources’ as a concept that incorporates three fundamental determinants of health, i.e., health literacy, social support, and self-efficacy, is novel as far as we know. This approach could be beneficial as it contributes to the understanding of the separate as well as the combined significance of these determinants for the health of refugee migrants in the early post-migration phase.

The aim of this study was to explore newly settled refugee migrants’ health literacy, social support, and self-efficacy and to investigate how these individual health factors were associated with general self-rated health (SRH) and psychological well-being.

## Material and methods

### Study design and setting

The study had a cross-sectional design and was carried out between October 2017 and March 2019 in seven selected cities in the Swedish county of Skåne. Data were extracted from a larger study aiming to evaluate the effect of Civic Orientation on participants’ health. We selected the locations for data collection in cooperation with the coordination office for the Civic Orientation within the County Administrative Board of Skåne to represent different geographical hubs; Malmö, Lund, Helsingborg, Kristianstad, and Ystad. Study questionnaires were distributed during the first session of Civic Orientation classes, which is part of the Introduction Program for newly settled migrants with a refugee background [34]. The program includes activities such as language training, vocational training, and Civic Orientation and has a duration of two years. Participation in the program is mandatory for obtaining welfare benefits.

### Study population

The study population consisted of newly settled adult migrants with a residence permit attending a Civic Orientation course. We used convenience sampling; the availability of classes in Arabic and availability of Arabic-speaking research staff to administer the paper and pencil questionnaire. The inclusion criteria in addition to being present were (1) enrolment in the Civic Orientation course, which meant all participant had a permanent or temporary residence permit, and (2) speaking Arabic. A team of Arabic speaking trained research assistants visited each course and informed the course participants about the study in their native language. Those who consented to participate were given the questionnaire that was filled out in the classroom.

We visited 61 classes. The Arabic-speaking research staff remained in the classrooms and assisted with reading the questions and answering questions for those who needed support. Out of 940 migrants present in the classrooms, 787 (84%) consented to participate in the study and were included in the analysis.

### Questionnaire

The questionnaire was a paper and pencil form. Three of the measures were already available in Arabic: General Self-Efficacy Scale (GSE) [35], GHQ-12 [30], and European Health Literacy Survey questionnaire 16 (HLS-EU-16) [36]. The remaining items were translated from Swedish to Arabic for the current project. The quality of the translations was reviewed by bilingual-speaking research personnel and checked in a back-translation, following the guidelines of scientific translations [37]. The complete questionnaire was then piloted on a group of earlier participants in the Civic Orientation.

### Independent variables

Independent variables were grouped into three sets: (1) health resources consisting of health literacy, emotional and practical social support, and self-efficacy; (2) sociodemographic variables including gender, age, education, type of residence permit; and (3) previous health (long-term illness).

*Health literacy* (HL) was assessed using the European Health Literacy Survey questionnaire 16 (HLS-EU-16) [38], which is a 16-item version of the original Health Literacy Scale-EU-47. HLS-EU-16 has been validated for general as well as migrant populations in Europe [38]. In the current study, the scale had a Cronbach alpha of 0.84. Each item is rated on a four-point scale (very easy, easy, difficult, very difficult). The ‘difficult’ options are assigned a value of 0 and the ‘easy’ options a value of 1, thus the possible range for the sum score is 0 to 16. For the purpose of analysis, the sum scores were dichotomized into sufficient HL (scores between 13–16) and limited HL (scores between 0–12) following the threshold reported for the HLS-EU-16 scale [38].

*The emotional and practical social support* questions were derived from the theoretical framework of social capital [24]. The questions have been theoretically and empirically validated in different settings [7, 39]. Emotional social support was phrased as “Do you have anybody whom you can share your deepest feelings with and confide in?” with no (0) and yes (1) response alternatives. Practical social support was phrased as “How many people in your surroundings can you easily ask for help with everyday tasks”, and dichotomized into none (0), and 1 or more persons (1).

*Self-efficacy* was measured using the General Self-Efficacy Scale (GSE), which is a 10-item scale measuring an individual’s belief in their ability to cope with and act on difficulties or situational demands [35]. The validity of the scale has been found satisfactory in several studies [40, 41]. In the current study, the scale had a Cronbach alpha of 0.87. Each item is reported on a four-point scale ranging from 1 to 4; not at all true (1), hardly true (2), moderately true (3), and completely true (4). Items were averaged to compute the total GSE score, which was then dichotomized into high and low (similar to HLS). It has been proposed that the mean of the general population can be used to determine the cut-off (usually found to be around 2.9) [35]. We used the mean score of our study population (= 3.0) as the cut-off.

The sociodemographic questions included gender, age, educational level, country of birth, type of residence permit (temporary or permanent), and year of receiving a residence permit. *Long-term illness* was measured by the question ‘‘Do you have any chronic disease or problems due to an accident or functional disability or other long-term health problem?” with no (0) and yes (1) response alternatives. This question is frequently used to assess self-reported long-term illness [42].

### Dependent variables

The two outcome measures in the study were self-rated health (SRH) and psychological well-being.

*Self-rated health* (SRH) was measured using the question “How do you assess your overall health status?” with five response alternatives: very good, good, neither good nor bad, bad, and very bad. This question measures general physical and emotional health. It is widely used and accepted as an indicator of a person’s subjective general perception of their health [3]. For the binary logistic regression analysis, we used a common dichotomization method [43, 44] and dichotomized the responses into good (the first two alternatives) and poor/less than good health (the last three alternatives).

*Psychological well-being* was measured using the 12-item version of the General Health Questionnaire (GHQ-12). The GHQ-12 has been extensively used in different countries and settings to assess the severity of non-psychotic mental health problems such as anxiety, depression, social dysfunction, and loss of confidence over the past few weeks [45]. The scale has been shown to have satisfactory validity and cross-cultural sensitivity [45, 46]. The Arabic version of the scale has been validated and used both in clinical and non-clinical settings [47, 48]. In the current study, the scale had a Cronbach alpha of 0.91.

Each item in the GHQ-12 has a four-point scale ranging from 0 (e.g., better than usual) to 3 (e.g., much less than usual), generating a sum score between 0 to 36 for each respondent. Higher scores indicate poorer conditions. The scores were dichotomized into good and poor psychological well-being, using a cut-off of 2/3 (meaning that scores above 12 were considered ‘poor/less than good’). Different thresholds for GHQ-12 are used, but 2/3 is a general cut-off point across different settings [45] and is commonly used in studies.

### Statistical analysis

The percentage of missing values across the 47 variables varied between 1% and 9.5% (missing at individual item level). Out of 787 records, 331 (42%) were incomplete. Older participants and those with lower education were more likely to have missing data. Missingness was also related to reporting long-term illness, poor general health, and lack of practical support. However, the later factors all relate to older age and lower education. Using multiple imputation, we created and analyzed 50 multiply imputed datasets. We used the default settings in SPSS 26 to impute the missing values at the item level under conditional specification. All the reported results are pooled unless specified otherwise.

We used descriptive statistics (numbers and percentages) to describe the characteristics of the study population (Table 1). Next, we used multivariate binary logistic analyses to investigate associations and change of impact of the independent variables on each of the two health outcome variables, when adjusted stepwise in three models. Initially, crude values were calculated for the main variables (Tables 2 and 3). In Model, 1 we adjusted for health resources (health literacy, emotional and practical social support, and self-efficacy). In Model 2, we added demographic variables (gender, age, educational level, and type of residence permit). In Model 3, we added previous health (long-term illness). Results are presented as odds ratios (OR), with 95% confidence interval. A *p*-value of <0.05 was considered statistically significant.

**Table 1:** Distribution of characteristics of study population by original and pooled data (n = 787).

	Original data			Pooled data	
Variable	Valid n (missing)	Number	%	Number	%
**Gender**	779 (8)				
Women		436	56.0	440.5	55.9
Men		343	44.0	346.5	44.1
**Age**	738 (49)				
19–29		231	31.3	240.0	30.5
30–49		415	56.2	446.0	56.7
50–69		92	12.5	101.0	12.8
**Education**	773 (14)				
0–6		124	16.0	126.1	16.0
7–9		165	21.3	169.5	21.5
10–12		203	26.3	207.7	26.4
More than 12		281	36.4	283.7	36.1
**Country of birth**	780 (7)				
Syria		575	73.7	579.4	73.6
Other		205	26.3	207.6	26.4
**Type of residence permit**	732 (55)				
Permanent		396	54.1	425.2	54.0
Temporary		336	45.9	361.8	46.0
**Long-term illness**	772 (15)				
No		532	68.9	543.0	69.0
Yes		240	31.1	244.0	31.0
**Health literacy**	586 (201)				
Sufficient		233	39.8	298.9	38.0
Limited		353	60.2	488.1	62.0
**Emotional social support**	769 (18)				
High		557	72.4	570.8	72.5
Low		212	27.6	216.2	27.5
**Practical social support**	765 (22)				
High		669	87.5	687.1	87.3
Low		96	12.5	99.9	12.7
**Self-efficacy**	669 (118)				
High		405	60.5	460.3	58.5
Low		264	39.5	326.7	41.5
**Self-rated health (SRH)**	775 (12)				
Good		500	65.4	504.6	64.1
Poor		275	35.5	282.4	35.9
**Psychological well-being**	704 (83)				
Good		473	67.2	520.6	66.1
Poor		231	32.8	266.4	33.9

**Table 2:** Logistic regression models explaining poor self-rated health (SRH) by individual-level resources, sociodemographic variables, and previous illness based on imputed data.

		Crude value			Model 1			Model 2			Model 3		
		OR	95% CI	*p*	OR	95% CI	*p*	OR	95% CI	*p*	OR	95% CI	*p*
**Individual resources**	**Health literacy**												
	Sufficient	ref			ref			ref					
	Limited	2.96	2.11–4.16	0.001	2.56	1.80–3.64	0.001	2.57	1.78–3.71	0.001	2.19	1.48–3.24	0.001
	**Emotional social support**												
	Yes	ref			ref			ref					
	No	1.83	1.32–2.53	0.001	1.3	0.91–1.87	0.150	1.58	1.07–2.32	0.021	1.59	1.05–2.42	0.030
	**Practical social support**												
	Yes	ref			ref			ref					
	No	1.92	1.24–2.95	0.003	1.56	0.97–2.50	0.064	1.58	0.97–2.59	0.069	1.89	1.12–3.20	0.017
	**Self-efficacy**												
	High	ref			ref			ref					
	Low	1.89	1.40–2.57	0.001	1.47	1.06–2.03	0.020	1.53	1.09–2.14	0.015	1.53	1.06–2.21	0.023
**Demographic**	**Gender**												
	Woman	ref						ref					
	Man	0.96	0.71–1.29	0.787				0.79	0.56–1.10	0.166	0.70	0.48–1.01	0.058
	**Age**												
	19–29	ref						ref					
	30–49	1.88	1.31–2.69	0.001				1.86	1.25–2.77	0.002	1.55	1.01–2.37	0.044
	50–69	4.63	2.76–7.76	0.001				5.98	3.38–10.59	0.001	3.40	1.81–6.39	0.001
	**Education**												
	0–6	ref						ref					
	7–9	0.92	0.58–1.48	0.739				1.10	0.66–1.84	0.727	1.31	0.75–2.31	0.341
	10–12	0.69	0.43–1.09	0.110				0.78	0.47–1.28	0.320	0.84	0.49–1.45	0.528
	More than 12	0.55	0.36–0.86	0.009				0.60	0.37–0.96	0.033	0.71	0.42–1.19	0.192
	**Type of residence permit**												
	Permanent	ref						ref					
	Temporary	0.83	0.61–1.13	0.235				0.99	0.69–1.42	0.970	1.10	0.75–1.62	0.625
**Previous illness**	**Long-term illness**												
	No	ref											
	Yes	7.13	5.06–10.04	0.001							6.09	4.18–8.86	0.001

Nagelkerke R Square (R^2^) Model 1 = 0.09–0.12, Model 2 = 0.17–0.21, Model 3 = 0.31–0.35.

OR = Odds ratio; CI = confidence interval; SRH was dichotomized into good and poor.

**Table 3:** Logistic regression models explaining poor psychological well-being (GHQ-12) by individual-level resources, sociodemographic variables and previous illness based on imputed data.

		Crude value			Model 1			Model 2			Model 3		
		OR	95% CI	*p*	OR	95% CI	*p*	OR	95% CI	*p*	OR	95% CI	*p*
**Individual resources**	**Health literacy**												
	Sufficient	ref			ref			ref			ref		
	Limited	3.64	2.53–5.23	0.001	2.80	1.91–4.10	0.001	2.84	1.92–4.20	0.001	2.68	1.80–3.98	0.001
	**Emotional social support**												
	Yes	ref			ref			ref			ref		
	No	2.95	2.12–4.09	0.001	2.07	1.44–2.99	0.001	2.14	1.46–3.15	0.001	2.13	1.45–3.14	0.001
	**Practical social support**												
	Yes	ref			ref			ref			ref		
	No	2.24	1.45–3.45	0.001	1.51	0.92–2.46	0.102	1.55	0.94–2.57	0.089	1.61	0.97–2.67	0.068
	**Self-efficacy**												
	High	ref			ref			ref			ref		
	Low	2.71	1.98–3.70	0.001	2.00	1.43–2.80	0.001	2.18	1.54–3.08	0.001	2.15	1.52–3.05	0.001
**Demographic**	**Gender**												
	Woman	ref						ref			ref		
	Man	1.28	0.94–1.72	0.109				1.14	0.81–1.61	0.460	1.12	0.79–1.58	0.533
	**Age**												
	19–29	ref						ref			ref		
	30–49	1.55	1.08–2.21	0.016				1.73	1.15–2.61	0.009	1.65	1.09–2.49	0.019
	50–69	2.31	1.39–3.84	0.001				3.32	1.85–5.96	0.001	2.76	1.50–5.06	0.001
	**Education**												
	0–6	ref						ref			ref		
	7–9	1.00	0.61–1.65	0.999				1.25	0.72–2.16	0.438	1.31	0.75–2.28	0.349
	10–12	0.85	0.52–1.38	0.509				0.99	0.58–1.69	0.959	1.02	0.60–1.76	0.935
	More than 12	1.34	0.85–2.12	0.204				1.73	1.05–2.84	0.032	1.85	1.12–3.08	0.017
	**Type of residence permit**												
	Permanent	ref						ref			ref		
	Temporary	1.21	0.89–1.64	0.226				1.25	0.87–1.80	0.222	1.29	0.90–1.86	0.172
**Previous illness**	**Long-term illness**												
	No	ref									ref		
	Yes	2.01	1.46–2.77	0.001							1.61	1.12–2.33	0.011

Nagelkerke R Square (R^2^) Model 1 = 0.17–0.19, Model 2 = 0.20–0.24, Model 3 = 0.21–0.25

OR = Odds ratio; CI = confidence interval; GHQ-12 was dichotomized into good and poor.

We used Statistical Package for the Social Sciences (SPSS), version 28.0, for all the statistical analyses.

### Ethical consideration

The participants received both oral and written information about the study in Arabic. The information included the aim of the study, the type of questions in the questionnaire, and how the data would be handled and stored. Participants were also informed that they could discontinue their participation at any time. Written informed consent was obtained from each participant prior to filling out the questionnaire. The study was approved by the Swedish Ethical Review Authority in Uppsala (registration number 2017/292).

## Results

### Sample characteristics

The sociodemographic characteristics and key variables in the study population are presented in Table 1. The original sample and the pooled data sets were very similar. The population consisted of more women (56%) than men. The average age was 36.3 years SD (10.6) and the median age was 35. The educational levels varied; 16% had no or little education (0–6 years), 48% had 7–12 years of education and 36% had more than 12 years of education (i.e., university or equivalent). Slightly more than half of the study population (54%) had a permanent residence permit. The vast majority of the participants (74%) were born in Syria. Based on the year of receiving the residence permit (not shown in the table) and the average handling time for asylum cases between 2016 and 2018 [49], the total time spent in Sweden at the time of the study was estimated to have been less than 3 years for the majority of the study participants.

The majority of participants had limited health literacy (60%), but high emotional and practical support (72% and 87% respectively) and high self-efficacy (60%).

The majority of the study population (65%) reported good or very good SRH, 23% reported neither good nor poor SRH, and 12% reported poor or very poor SRH. The majority reported good psychological well-being (67%). The mean score for psychological well-being (GHQ-12) was 11.14 (SD = 7.04) in the population and the median was 10.

### Logistic regression models

Tables 2 and 3 show the results from the multivariate binary logistic regression for SRH and psychological well-being respectively. In the adjusted logistic regression models, the pattern of results was very similar for both outcomes. Lack of health resources i.e., limited health literacy, lack of emotional social support, and low self-efficacy were consistently associated with poor health outcomes. Limited health literacy had the strongest statistically significant association with poor SRH (odds ratio (OR) = 2.19, 95% confidence interval (CI) = 1.48–3.24) followed by no practical social support (OR = 1.89, 95% CI = 1.12–3.20). Limited health literacy also had the strongest statistically significant association with poor psychological well-being (OR = 2.68, 95% CI = 1.80–3.98), followed by low self-efficacy (OR = 2.15, 95% CI = 1.52–3.05).

Among the demographic variables, older age was associated with poor SRH. Older age and higher education (more than 12 years) were associated with poor psychological well-being. Type of residence permit was not associated with either health outcome.

Previous long-term illness was associated with poor SRH (OR = 6.09, 95% CI = 4.18–8.86) and with poor psychological well-being (OR = 1.61, 95% CI = 1.12–2.33).

## Discussion

The aim of this study was to explore newly settled migrants’ health from a number of individual health-related factors, i.e., health literacy, emotional and practical social support, self-efficacy, and sociodemographic characteristics. The analyses showed that lack of health resources i.e., limited health literacy, lack of emotional support, and low self-efficacy were consistently associated with reporting poor general self-rated health and poor psychological well-being. However, demographic variables were not as imperative in relation to the other included variables. The results point to the importance of individual health resources for a comprehensive understanding of newly settled refugee migrants’ health.

The results regarding limited health literacy and poor SRH and psychological well-being are in line with previous studies on health literacy in general populations [42] and among newly settled migrants [29, 30]. Moreover, the distribution of limited health literacy in our sample (60%) is similar to that found in other studies on similar populations [29, 30]. Theoretical models have been suggested and empirical studies have shown that limited health literacy is linked to poor utilization of health care [22, 30], which could explain the relatively strong association between health literacy and health outcomes found in our study. The suboptimal use of health care is, in turn, affected by structural hinders such as lack of health information and limited access to digital technology [19]. Our findings add to growing evidence of the importance of health literacy for migrants’ health and the need to address health literacy in health promotion efforts. Health literacy, strengthened through tailored educational programs has been suggested as a means to promote health and empowerment among individuals [19, 50]. In the Swedish context of early post-migration, adaptations of clinical practices could be beneficial, for example, changing medical examinations for asylum seekers to accommodate those with limited health literacy [36]. Another possibility would be to adapt and expand the health information communicated in Civic Orientation courses [51].

The association between personal levels of low social support and poor SRH and psychological well-being has been shown in studies on general populations [23, 52] and migrant populations [33, 53] as well as in comparative studies including both groups [7]. In a Swedish study, social capital was found to mediate associations between weak socioeconomic position and discrimination, and poor mental health among newly settled migrants [33]. In the same study, social support had the strongest mediatory role on health outcomes, albeit augmented by other types of social capital.

In our study, we observed a significant association between practical support and SRH but not psychological well-being. In our study, we observed a significant association between practical support and SRH but not psychological well-being. This could imply a real difference between practical social support and SRH and psychological well-being, but it could also be an effect of the skewness of the variable [54]. Nevertheless, it is evident from our results and other studies that social support is important for newly settled refugee migrants’ health and that lack of it needs to be addressed [26, 33]. Increased social participation at both individual and community levels could increase migrants’ access to social resources [24, 33] and strengthen their resilience against challenges in the post-migration phase. Co-produced interventions based on specific social needs could be a way [55]. The engagement of civil society is also important for empowering migrants socially through community-based local interactions [56].

The relation between low self-efficacy and poor psychological well-being found in our study corroborates findings from other studies on migrant populations, focusing both on migrants with trauma-related symptoms [57] and general migrant populations [32, 58]. The level of self-efficacy in our study was similar to that reported in an Australian study on newly settled migrants [32]. In addition, they found that newly settled migrants reported a significantly lower self-efficacy compared with those resettled for 10 years or more, which highlights the relevance of focusing on the early post-migration phase. However, a recent study on a general population of newly resettled migrants found no relation between self-efficacy and mental health [59]. A common feature of the studies on migrant populations and self-efficacy is that they are performed on relatively small samples (< 200) [32, 57–59], which makes generalizations difficult and suggest that more research in larger and more heterogeneous migrant populations is needed. Nevertheless, for the newly settled migrants, believing in their ability to cope with and act on individual and structural challenges in the demanding resettlement phase could be a key resource as it is linked to and complements all other health resources [25].

In our study, education was not significantly associated with poor SRH and psychological well-being in crude values nor when we controlled for other variables in the regression models. There was only one exception; having 12 years of education or more was associated with poor psychological well-being. This finding contradicts theoretical assumptions and empirical studies on the protective role of higher education on health. However, for migrants, the pattern might differ. A literature review assessing the prevalence of psychological disorders among war-affected refugees reports associations between demographic factors (age, gender, and education) and psychological disorders in univariate analysis but not once adjusted for confounders in multivariate analysis [6]. Additionally, a Swedish study on mental health among Syrian refugee migrants showed that mental ill health does not appear to differ by educational level [60]. Possible explanations are that migrants with the highest education must reconcile with a greater loss of status, poorer employment quality and over-qualification [60, 61], and an added stress of finding employment compatible with their high education. Overall, results on the association between educational level and mental health among refugees are contradictory, suggesting differences among the refugee migrant populations and that other post-migration factors (e.g., socioeconomic status, social support) might be stronger predictors of mental health among migrants.

Our study found no significant associations between the type of residence permit and health outcomes. Earlier studies point to the detrimental effects of restrictive immigration policies on psychological health and well-being [11, 62]. However, most of the reviewed studies have been performed in detention settings or in settings where temporary residence permits entail more restrictions compared to Sweden such as limited access to services and health care [62–64]. The results could also reflect that the total burden of post-migration stressors and barriers e.g., socioeconomic disadvantage, inadequate housing, limited employment opportunities, and experiences of discrimination supersede the effect of temporary residence permits on health.

In summary, our study confirms earlier studies on migrant populations that link limited health literacy, low social support, and low self-efficacy to poor self-rated and mental health and adds understanding about these factors’ influence on the health of newly settled migrants. Importantly, these individual-level resources (i.e., downstream and midstream social determinants of health) are not created by the individual alone. Rather they are shaped by upstream determinants such as the distribution of economic and social resources, opportunities, and policies [20]. When creating policies and interventions aimed at newly settled refugee migrants, health resources such as health literacy, social capital and self-efficacy should therefore be considered. The mandated introduction activities such as language training and civic orientation reach a large number of migrants and could be used as an arena to implement needed health interventions.

### Strengths and limitations

Our study had a relatively large sample (n = 787) that we believe was facilitated by administering the questionnaire within the civic orientation classes. Another strength was that we measured both outcomes and independent variables using standardized tools that have been previously used and validated across languages and cultures. This is essential for increasing comparability and replicability.

The survey was administered by research assistants and language support personnel with a similar backgrounds and shared language with the respondents. Based on our experience, this approach to data collection lessens the formal nature of the exercise and promotes a sense of safety, which facilitates participation as it enables potential respondents to ask questions about the study and what participation entails. Additionally, it was stressed both orally and in writing that participation was voluntary and that non-participation would not affect their residence permit status in any way. Nevertheless, as respondents were part of a mandated introduction program, some may have felt obliged to participate. Ethically this would be problematic because it may contribute to a feeling of vulnerability and stress for respondents. As for implication on the quality of data, we do not expect large inferences on the outcome measurements, as it is not evident what the “right” or expected answer would be.

Arabic speaking personnel were available in the classrooms to assist in answering questions about the survey and reading questions for those who needed support. Nevertheless, it cannot be ruled out that a higher proportion of individuals with fewer years of education opted out of participation as sociodemographic characteristics are known to influence participation in survey studies [65]. Our population consisted of only Arabic- speaking newly settled migrants, predominantly from Syria, and other countries in the Middle East affected by war. Our population was also homogenous in terms of time spent in Sweden (< 5 years) and level of income since participants in the Civic Orientation receive standardized welfare benefits or salaries while in the Introduction Program. This made stratifications according to country of origin and income redundant and therefore we could not examine the potential impact of country of origin and income in this study.

Another limitation is that in a cross-sectional design, there is a risk of reverse causality. In our study, there is a risk that cause effect relation between health literacy, social support, and self-efficacy, and health outcomes could be reversed. Moreover, we could not control for pre-migration circumstances, e.g., exposure to trauma, due to lack of data, which otherwise could have eliminated the potential effect of psychological distress on the studied health resources. However, even though the mental health of newly settled refugee migrants may be negatively impacted by traumatic events, the long-term effects of trauma on the mental health of migrants are debated [10, 66].

As for health literacy, it is theorized that limited health literacy can be an explanatory factor for ill health through the pathway of behaviors, such as refraining from seeking health care [22, 67]. Even though the pathway is plausible in the case of newly settled migrants, the reverse cannot be entirely ruled out [22]. Similarly, social support has been theorized to have a causal effect on health via different need and support mechanisms, moderating stress and promoting coping [68] but also linked to several health outcomes in empirical studies [23, 69].

The correlation between self-efficacy and health has been described as one intercepted by exposure to stressful events that appear uncontrollable to the individual. Low self-efficacy, i.e., low perceived ability to cope, prevents individuals from effectively dealing with these stressful events and is believed to create biological responses that lead to ill health [25]. However, the reverse could also be true as an individual’s physiological and mental state affect how they rate their self-efficacy [25].

## Conclusions

Our results suggest that health literacy, emotional social support, and self-efficacy may have an important role in the general health and psychological well-being of newly settled migrants. Health literacy and social support are modifiable factors that can be addressed through the introduction policies and activities as well as health intervention programs for newly settled refugee migrants. If considered and strengthened in the early post-migration phase, these health resources can create resilience and buffer against challenges experienced in the establishment phase, which in turn could positively influence future health and social integration.
